# An Unusual Case of Severe Atelectasis: Mucus Impaction in a Young Obese Female

**DOI:** 10.7759/cureus.49932

**Published:** 2023-12-04

**Authors:** Owen Cole, Nishant Patel

**Affiliations:** 1 Internal Medicine, The Brooklyn Hospital Center, New York, USA

**Keywords:** chest x-ray, bronchoscopy, pulmonary disease, respiratory distress, atelectasis

## Abstract

Atelectasis is defined as the the loss of lung volume due to collapse of lung tissue and is usually associated with symptoms of respiratory distress, including increased work of breathing and increased oxygen requirements. It is common in hospitalized patients with limited mobility and in patients with underlying lung conditions. Treatment is largely supportive when no underlying condition is identified. It is rare to occur in otherwise healthy individuals. The patient in this case presented to the emergency department with complaints of progressive shortness of breath, productive cough, chest tightness, subjective fever, chills, and nasal congestion for two weeks. Physical exam revealed decreased breath sounds on the left side, raising the suspicion for atelectasis or pneumothorax. Chest X-ray revealed complete white-out of the left lung. Bronchoscopy was performed and revealed mucus impaction in the left mainstem bronchus, which was removed. Repeat chest X-ray revealed resolution of atelectasis. The patient’s symptoms improved, and she was discharged with outpatient pulmonary follow-up. The case described below illustrates that even in young patients with no underlying comorbidities, other than obesity, atelectasis as a cause of respiratory complaints should always be considered.

## Introduction

Atelectasis describes the loss of lung volume due to collapse of lung tissue and is usually associated with symptoms of respiratory distress, including increased work of breathing and increased oxygen requirements [1,2]. It commonly occurs in hospitalized patients who have limited mobility, especially during post-procedure courses, and patients with underlying lung pathologies [3,4]. Atelectasis can occur in patients who present asymptomatically and should be suspected when chest radiography reveals areas of a collapsed, white-out lung [3]. Patients may also present with complaints of shortness of breath and signs of respiratory distress. Treatment is largely supportive in cases when no underlying cause can be identified. Obesity is known to cause restrictive lung disease and increases the risk of post-operative pulmonary complications, including atelectasis [5,6,7]. However, the research into obesity as a risk factor for atelectasis outside the perioperative period is limited [6,7]. The case described below suggests that in young obese patients with no underlying lung pathology, atelectasis as a cause of respiratory complaints should always be considered.

## Case presentation

A 29-year-old obese (BMI 44.8) African American female with no known past medical history presented to the emergency department with complaints of progressive shortness of breath, productive cough, chest tightness, subjective fever, chills, and nasal congestion for two weeks. She denied any tobacco, alcohol, or recreational drug use. She denied any prior chest imaging or medical diagnoses, including underlying lung pathology. The patient reported that she has had a decrease in exercise tolerance and now has shortness of breath after walking half a flight of stairs; she previously would get short of breath after one flight of stairs. The patient also reported developing an upper respiratory tract infection two weeks ago. She reported productive yellowish sputum that is decreasing in quantity and is not foul smelling or blood-tinged. The patient went to an urgent care clinic where she tested negative for influenza and COVID. A chest X-ray done at urgent care revealed complete white-out of the left lung, suggesting left lung atelectasis, and she was sent to the emergency department. 

**Figure 1:**
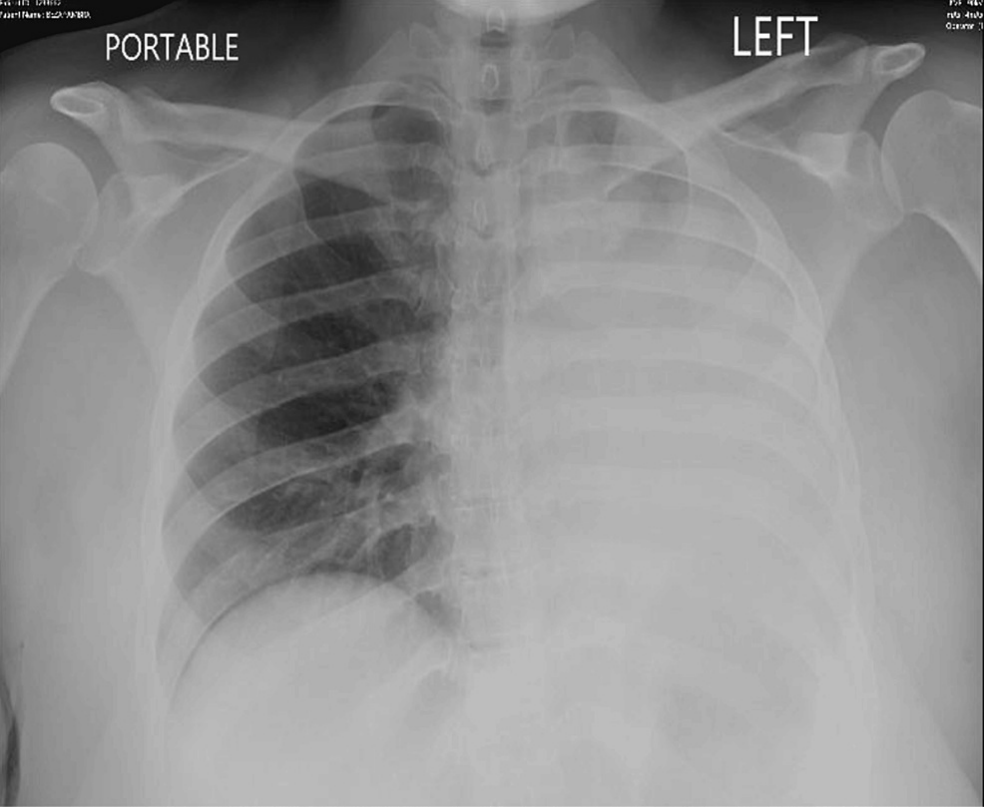
Chest X-ray on admission, prior to bronchoscopy

**Figure 2:**
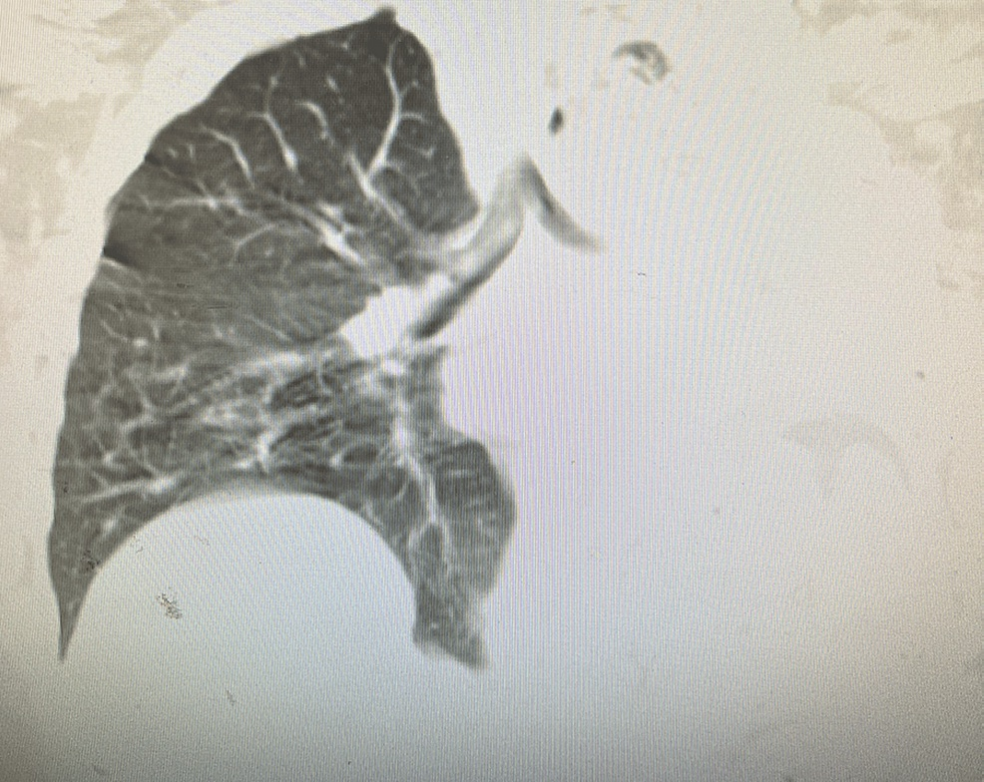
CT chest without contrast done on the day of admission

On presentation at the emergency department, she was afebrile, saturating above 96% on room air, but tachypneic to 28 breaths/minute. Initial laboratory evaluation was unremarkable, without leukocytosis. Arterial blood gas done with pH 7.47 CO_2_ 30 mol/L and HCO_3_ 22 nmol/L. Physical exam revealed decreased breath sounds on the left side, with some breath sounds auscultated on the left apical zone. The right lung was clear to auscultation. There was no lower limb edema, and the calves were nontender bilaterally. There was suspicion for pneumothorax, severe atelectasis, or hemothorax at this point. A chest X-ray at admission (Figure 1) revealed a near-complete opacification of the left lung with some apical aeration and leftward tracheal deviation. A noncontrast chest CT was done on the day of admission, which showed a complete collapse of the left lower lobe, minimal aeration of the apical portion of the upper lobe, and abrupt termination of the left mainstem bronchus (Figure 2). This confirmed the diagnosis of severe atelectasis. An arterial blood gas revealed mild respiratory alkalosis. The patient was retested for COVID and influenza, methicillin-resistant *Staphylococcus aureus* (MRSA), and *Legionella*, which were negative. Blood cultures were negative, and respiratory culture revealed normal flora.

**Figure 3:**
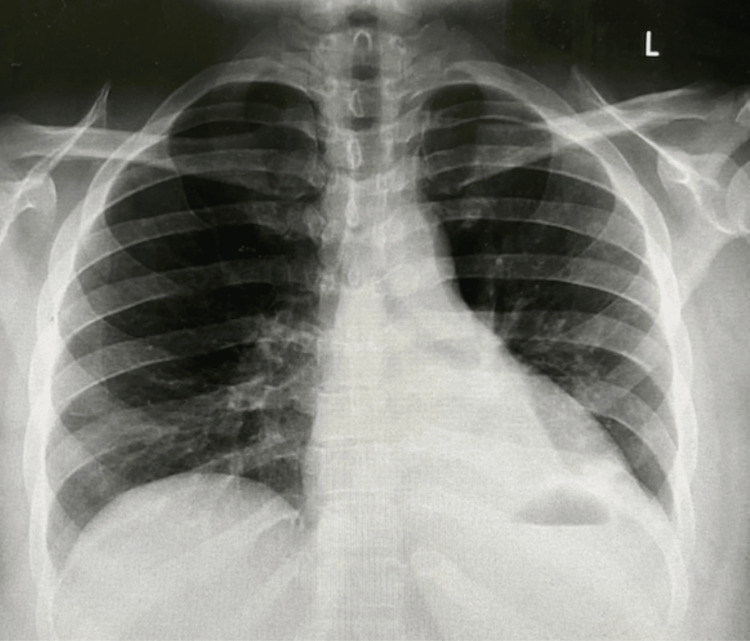
Chest X-ray after bronchoscopy six days after admission

The patient was initially treated with albuterol-ipratropium nebulizer, inhaled hypertonic saline, inhaled acetylcysteine, incentive spirometry, and aggressive chest physical therapy. The patient was also started on community-acquired pneumonia coverage with ceftriaxone and azithromycin for presumed post-obstructive pneumonia. A repeat chest X-ray on the second day of admission revealed marked improvements in the upper left lobe, with persistent collapse of the left lower lobe. Despite a positive response to the treatment, there was concern for endobronchial stenosis from a variety of possible causes, and thus a bronchoscopy was warranted for a direct visualization of the airways. Bronchoscopy revealed a normal-appearing right mainstem bronchus. Upon evaluation of the left main stem bronchus, thick white mucus was visualized that was not easily suctioned. After repeated suctioning attempts, the mucoid secretions were removed, and the segments of the left bronchus revealed no abnormalities. The mucoid samples were sent for cytology studies. Upon cytologic and microbiological analysis, the samples revealed normal respiratory flora, normal ciliated columnar epithelium, histiocytes, and inflammatory cells. It was negative for malignancy, acid fast bacilli, fungi, and pathologic bacteria and viruses. A diagnosis was made of the left mainstem bronchus mucoid impaction causing compressive atelectasis. Continued management with albuterol-ipratropium nebulizer, inhaled hypertonic saline, incentive spirometry, and chest physical therapy was warranted until there is radiographic evidence of resolution of the left-lower-lobe atelectasis. Repeat chest X-ray done six days later after medical management and bronchoscopy showed reinflation of the left lung with resolution of the atelectasis (Figure 3). The patient was discharged with outpatient pulmonary follow-up. She is currently pending pulmonary function tests and polysomnography to evaluate for possible obesity hypoventilation syndrome and/or obstructive sleep apnea as a contributing risk factor to her presentation. These studies were not obtained during hospitalization as they are not regularly performed at the institution on an inpatient basis. 

## Discussion

An imaging finding on chest X-ray of a small, opacified ipsilateral hemithorax associated with a mediastinal shift to the ipsilateral side, such as in this case, is suspicious for atelectasis [1]. Other causes include large plural effusion, hemothorax, or prior pneumonectomy. There are multiple causes for atelectasis, among which, external compression, absorption of the intra-alveolar air, and impairment of the surfactant function are the common ones [2]. Resorption atelectasis (also called gas atelectasis) occurs when a pocket of gas, created distal to a complete airway occlusion, gets absorbed, causing atelectasis. Distal to the complete airway occlusion, lung areas with a very particular ventilation-perfusion mismatch occurs where perfusion is intact but ventilation is cut off, resulting in greater rates of gas absorption than ventilation, leading to atelectasis [2]. Body habitus is also a great contributor to development of atelectasis. Obesity, as in the presented case above, promotes airway closing to a greater extent than in healthy-weight adults due to low functional reserve capacity (FRC) [2]. Conventional chest radiography features suggestive of atelectasis includes signs of volume loss on ipsilateral lung zones and contralateral hyperinflation. Signs of volume loss include mediastinal shift, hemidiaphragm elevation, and displacement of interlobar fissure. Diagnosis of the cause of atelectasis based on radiographic findings involves clinical correlation with presenting symptoms in view of medical history and comorbidities, which makes it difficult to prospectively diagnose in young patients with no prior medical history or classical risk factors, such as in this patient [2]. Initial concerns for atelectasis are raised by chest radiographs, while CT scans remain the definitive tests for diagnosis. Bedside ultrasound could also help distinguish between pleural fluids and primary lung collapse, but distinguishing signs are often subtle.

Atelectasis can be broken down pathophysiologically into obstructive and nonobstructive atelectasis. Obstructive atelectasis occurs due to a blockage in the airway. When air distal to the blockage is resorbed, the lung becomes gasless and collapses. This is commonly seen with tumors or masses that compress the airway or in children who aspirate foreign objects [2]. Nonobstructive atelectasis can be caused by the loss of contact between the parietal and visceral pleura, lung parenchyma compression, surfactant dysfunction, and replacement of lung tissue by scarring or infiltrative disease and by strong vertical acceleration forces. Relaxation atelectasis occurs when contact between the visceral and parietal pleura is lost usually due to pleural effusion or pneumothorax and occurs because of the elastic recoil of lung parenchyma. Compressive atelectasis occurs due to the compressive forces of a space occupying lesion in the thorax. Adhesive atelectasis is due to surfactant dysfunction and is commonly seen in acute respiratory distress syndrome in adults or respiratory distress syndrome of the newborn. Replacement atelectasis occurs when the aveoli of an entire lobe are filled by a tumor causing loss of lung volume. Cicatricial atelectasis is the loss the lung volume due to scarring. Acceleration atelectasis has been seen in pilots who experience high vertical acceleration forces causing loss of lung volume. Rounded atelectasis, also known as folded lung, occurs due to asbestosis related pleural disease. Plate-like atelectasis, also known as subsegmental, discoid, or linear, is a very common cause of temporary atelectasis occurring in regions of the lung that are poorly ventilated, close to linear scars, or adjacent to pulmonary fissures [3,4]. 

The patient described in this case was diagnosed with obstructive atelectasis due to mucus impaction, likely in the setting of a recent upper respiratory tract infection. Bronchoscopy confirmed the cause of obstruction as an impacted mucus plug in the left mainstem bronchus causing resorptive atelectasis and the subsequent symptoms of respiratory distress. After a direct removal of the mucus plug, the patient's symptoms improved, and she was discharged with outpatient follow-up. It highlights the importance of considering atelectasis as a cause of respiratory distress in patients who present with shortness of breath and with physical exam findings of decreased breath sounds on auscultation. It can be managed with hypertonic saline, inhaled acetylcysteine, chest percussion therapy, inhaled bronchodilators, increased mobility, and getting patients out of bed and mobile. When these measures fail, direct visualization with bronchoscopy and possible removal of impaction is required. It is imperative to have a direct visualization of the airway to look for neoplasms, foreign bodies, or other anomalies causing obstruction and resorptive atelectasis. This patient warranted bronchoscopy in order to rule out airway abnormalities, but was ultimately diagnosed with an impacted mucus plug. The patient also needed to had official pulmonary function tests and polysomnography to evaluate for obesity hypoventilation syndrome and/or obstructive sleep apnea as potential contributing factors to her presentation. 

Obesity is a known contributor to multiple lung pathologies, including obstructive sleep apnea, obesity hypoventilation syndrome, and restrive lung disease [5]. Obesity causes reduced lung volumes and contributes to airway collapse during exhalation [5]. Obese patients are also at greater risk for postoperative pulmonary complications, including postoperative atelectasis [6,7]. Obesity contributes to significant postoperative atelectasis and is considered an independent risk factor for postoperative atelectasis owing to decreased functional residual capacity [7]. Upper respiratory tract infections are also known to contribute to postoperative atelectasis [8]. Despite obesity and upper respiratory tract infections being known contributors to postoperative pulmonary complications, the research into obesity as a risk factor for atelectasis outside the perioperative period is limited. Additional research into obesity and obesity related lung conditions and their association with atelectasis especially after upper respiratory tract infections is needed to quantify these risks. The only contributing factors to this patient's atelectasis were obesity and a recent upper respiratory tract infection. This case shows the fact that, although rare, atelectasis can occur in obese patients who present with respiratory distress.

## Conclusions

Atelectasis is defined as the the loss of lung volume due to collapse of lung tissue and is usually associated with symptoms of respiratory distress, including increased work of breathing and increased oxygen requirements. It is common in hospitalized patients with limited mobility and in patients with underlying lung conditions. Treatment is largely supportive when no underlying condition is identified. It is rare to occur in otherwise healthy individuals. There is little literature on spontaneous complete lung atelectasis in otherwise healthy individuals. The management of atelectasis is dependent on the underlying cause but is largely supportive. It is important to rule out bronchial neoplasms and foreign bodies via bronchoscopy when patients present with complete atelectasis. Obesity is a known contributor to both pulmonary disease and post-operative atelectasis. Upper respiratory tract infections are also known to increase the risk of postoperative atelectasis. This case illustrates a rare presentation of an impacted mucus plug in a young obese adult with no history of recent immobilization. It demonstrates the importance of considering a diagnosis of atelectasis in obese patients who present with respiratory distress especially in the setting of recent upper respiratory tract infections.
